# Identification and Characterization of a Novel Quanzhou Mulberry Virus from Mulberry (*Morus alba*)

**DOI:** 10.3390/v15051131

**Published:** 2023-05-09

**Authors:** Jia Wei, Lei Chen, Zilong Xu, Peigang Liu, Yan Zhu, Tianbao Lin, Lu Yang, Yuan Huang, Zhiqiang Lv

**Affiliations:** 1 Institute of Sericulture and Tea, Zhejiang Academy of Agricultural Sciences, Hangzhou 311251, China; chenlei_0117@163.com (L.C.); 18726089100@163.com (Z.X.); liupeigang@zaas.ac.cn (P.L.); zhuy@zaas.ac.cn (Y.Z.); kissumbra@126.com (T.L.); hy18297698386@163.com (Y.H.); 13958131715@139.com (Z.L.); 2 Key Laboratory of Forest Resources and Utilization in Xinjiang of National Forestry and Grassland Administration, Urumqi 830052, China; yanglukitty127@163.com; 3 Key Laboratory of Fruit Tree Species Breeding and Cultivation in Xinjiang, Urumqi 830052, China; 4 School of Life Sciences, Anhui Agricultural University, Hefei 230036, China

**Keywords:** *Morus alba*, Quanzhou mulberry virus, transcriptome sequencing, unclassified Riboviria, virus infection

## Abstract

In this study, we discovered a new virus named Quanzhou mulberry virus (QMV), which was identified from the leaves of an ancient mulberry tree. This tree is over 1300 years old and is located at Fujian Kaiyuan Temple, a renowned cultural heritage site in China. We obtained the complete genome sequence of QMV using RNA sequencing followed by rapid amplification of complementary DNA ends (RACE). The QMV genome is 9256 nucleotides (nt) long and encodes five open reading frames (ORFs). Its virion was made of icosahedral particles. Phylogenetic analysis suggests that it belongs to the unclassified Riboviria. An infectious clone for QMV was generated and agroinfiltrated into *Nicotiana benthamiana* and mulberry, resulting in no visible disease symptoms. However, systemic movement of the virus was only observed in mulberry seedlings, suggesting that it has a host-specific pattern of movement. Our findings provide a valuable reference for further studies on QMV and related viruses, contributing to the understanding of viral evolution and biodiversity in mulberry.

## 1. Introduction

Mulberry (*Morus alba*) is a deciduous tree belonging to the genus *Morus* in the family Moraceae and has been cultivated in China for centuries due to its economic significance [1]. Its leaves serve as food for silkworms [2], and its fruits can be consumed directly or processed into jam, juice, and desserts [3,4,5,6]. However, mulberry viruses that impact the yield and quality of both mulberry leaves and fruits have been discovered and reported in recent years. Mulberry crinkle leaf virus (MCLV) [7], mulberry mosaic dwarf-associated virus (MMDaV) [8], mulberry cryptic virus 1 (MuCV1) [9], and citrus leaf blotch virus-ML (CLBV-ML) [10] all cause mosaic symptoms in mulberry trees. Mulberry badnavirus 1 (MBV1) [11] affects both leaf and fruit development. In contrast, there are also some viruses present in mulberry trees that do not cause significant damage due to their low pathogenic ability and low accumulation, such as Citrus leaf blotch virus isolate mulberry alba 2 (CLBV-ML2) and Mulberry-associated virga-like virus (MaVLV) [12]. It is still essential to identify and characterize these viruses to better understand their potential impact on the crop.

Compared to highly pathogenic viruses that cause noticeable symptoms, there has been relatively less research on plant viruses with low pathogenicity and subtle symptoms. However, studying these types of viruses is meaningful and has practical applications. For example, viral cross-protection has been used to protect crops such as tomatoes, cucumbers, and potatoes from severe strains of viruses through pre-immunization using mild strains of viruses [13,14,15,16,17]. Furthermore, citrus leaf blotch virus-based vectors have been successfully used to express foreign genes in citrus without the need for transformation and regeneration techniques required to obtain transgenic plants [18]. Such viruses should cause symptomless infections to ensure that phenotype changes due to endogenous gene silencing or foreign gene expression are not masked by virus symptoms.

Recently, several symptomless viruses have been reported in various crops, including Blueberry latent virus (BBLV) in blueberry (*Vaccinium corymbosum*) [19], Persimmon cryptic virus (PeCV) in Japanese persimmon (*Diospyros kaki*) [20], and Arhar cryptic virus-1 (ArCV-1) extracted from asymptomatic pigeonpea plants (*Cajanus cajan*) [21]. In the case of mulberry trees, their unique features make them valuable for identifying and characterizing new viruses, and old mulberry trees are particularly significant for this purpose due to their longevity. In our previous studies, transcriptome sequencing technology was utilized to identify three viruses from eight old mulberry trees [12]. Therefore, we employed the same technology to analyze a sample collected from an ancient mulberry tree over 1300 years old located at Fujian Kaiyuan Temple in Quanzhou, Fujian Province. A novel virus has been discovered with no homology to any known virus. Further investigation suggested that the virus can infect both *N. benthamiana* and mulberry, but systemic movement was only observed in mulberry seedlings. This host-specific movement feature of QMV suggests that it may have evolved to specifically infect and move within mulberry plants, making it a potential candidate for targeted delivery of transgenes or for use as a biological control agent against mulberry pests or diseases. In addition, further research is needed to explore the molecular mechanisms behind QMV’s host-specific movement and to investigate its potential as a tool for genetic modification and disease management in the mulberry industry.

## 2. Materials and Methods

### 2.1. Plant Material

The present study examined mulberry leaves that were collected from Fujian Kaiyuan temple, Quanzhou, Fujian Province, China (118°58′ N, 24°90′ E), which were asymptomatic at the time of collection. Following their removal, the leaves were immediately placed in liquid nitrogen for rapid freezing and then stored at −80 °C until further analysis.

### 2.2. RNA Extraction and Sequence Analyses

The sample underwent transcriptome sequencing and analysis at Majorbio Corporation (located in Shanghai, China). A standard non-strand-specific cDNA library was prepared, followed, and subjected to RNA sequencing (paired-end, 150 bp) on an Illumina HiSeq 2500 platform (Illumina, San Diego, CA, USA). Raw data trimming and contig assembly were conducted using Trimmomatic (Version 3.90) [22] and Trinity (Version 2.8.5) [23] with default parameters. The assembled contigs were subjected to a Blastx search using the BLAST suite web tool to identify viral proteins [24]. The viral open reading frames (ORFs) were predicted using the ORF-finder web tool (https://www.ncbi.nlm.nih.gov/orffinder/, accessed on 10 January 2023), and conserved domains were searched using the CDD/SPARCLE web tool (https://www.ncbi.nlm.nih.gov/Structure/cdd/wrpsb.cgi/, accessed on 10 January 2023) provided by NCBI (Bethesda, MD, USA) [25].

### 2.3. Full-Length Genome Amplification, Sequencing

Total RNA was extracted from leaf tissues using the MiniBEST Universal RNA Extraction Kit (purchased from Takara, Beijing, China), and the complete genome of QMV was obtained through rapid amplification of cDNA ends (RACE) technology using the SMARTer^®^ RACE 5′/3′ kit (purchased from Takara, Beijing, China).

The kit-specific primers were used to synthesize cDNAs for amplification of the 5′ and 3′ end sequences. Amplification was performed using Hieff Canace^®^ Plus High-Fidelity DNA Polymerase (purchased from Yeasen, Shanghai, China), along with a universal primer mix (UPM, CTAATACGACTCACTATAGGGCAAGCAGTGGTATCAACGCAGAGT) and gene-specific primers (GSP, QMV-5UTR-R: GATTACGCCAAGCTTGAGATATTTTGCGGTGATACG; QMV-5UTR-R1: CCCGTTCTCCCACTCTTTGTCCTGCTTC; QMV-3UTR-F: GATTACGCCAAGCTTGACAGTCTACCACTACTCAATC; QMV-3UTR-F1: GAGCCAGGAATCGGTTTTTACCGGGGGT) for the unknown 5′ and 3′-terminal sequences. The purified 5′ and 3′-terminal products were concatenated with a pESI-T vector (purchased from Yeasen, Shanghai, China), followed by transformation into DH5α competent cells. Positive clones were identified via colony PCR using detection primers (M13-F: CAGGGTTTTCCCAGTCACG; M13-R: GAGCGGATAACAATTTCACAC) and sequenced at Youkang Corporation (located in Hangzhou, China). Finally, the full-length genome sequence was assembled using DNAMAN software (version 9.0).

### 2.4. Phylogenetic Analyses

In this study, we performed phylogenetic analysis using the ORF1 and ORF2 amino acid sequences from the QMV full-length genomic sequence (File S1). These sequences, which were used for phylogenetic trees, were downloaded from the NCBI website and compared with QMV ORF1 and ORF2 sequences by ClustalW (Clustal W 1.81, Dublin, Ireland) [26]. Conservative regions of the sequences were selected and cut using Gblock (G-Block, Wallonia, Belgium) and trimAI software (trimAI v1.2, Barcelona, Spain). The optimum model for phylogenetic tree building was selected by ModelFinder software (implemented in IQ-TREE version 1.6.12). Finally, the maximum likelihood (ML) algorithm with PMB and JTTDCMut substitution models were used to construct phylogenetic trees in IQ-TREE (version 1.6.12) with 1000 bootstrap replications [27].

### 2.5. Construction of a QMV Infectious Clone and Agroinfiltration

To construct a QMV infectious clone, two primer pairs (pCB301-QMV-F1: CAGGTCGACTCTAGAGGATCACATGGGTTTACCAATACATTATC; pCB301-QMV-R1: CGTCGATGAAAAGAGTCTGACAAGATGGAGCACC; pCB301-QMV-F2: TCAGACTCTTTTCATCGACGAGTGTCTTATGTAT; pCB301-QMV-R2: TGAACGATCGGGGAAATTCGCCAATGCATAACTAAACGGAAGGAAG) with homology arms were designed using the Takara primer design website (https://www.takarabio.com/learning-centers/cloning/primer-design-and-other-tools/, accessed on 15 February 2023). Two fragments covering the full-length genomic RNA of QMV were amplified and inserted into the pCB301 vector (gifted from Prof. Zongtao Sun, School of Marine Sciences, NingBo University) using the seamless cloning technique.

Total RNA was extracted from leaf tissues using the MiniBEST Universal RNA Extraction Kit (purchased from Takara, Beijing, China) and reverse transcribed into cDNA using Hifair^®^ III 1st Strand cDNA Synthesis Kit (purchased from Yeasen, Shanghai, China). PCR amplifications were performed with Hieff Canace^®^ Plus High-Fidelity DNA Polymerase (purchased from Yeasen, Shanghai, China) using a reaction mixture containing 25 μL 2×Canace^®^ Plus PCR buffer, 1 μL cDNA, 2 μL forward/reverse primer (10 μM), 1 μL Hieff Canace^®^ Plus High-Fidelity DNA Polymerase (1 U/μL), and 19 μL distilled water. The PCR amplification procedure was as follows: (1) 3 min denaturation at 98 °C; (2) 30 cycles, each consisting of 10 s at 98 °C, 20 s at 65 °C, and 2.5 min at 72 °C; and (3) 5 min final extension at 72 °C. The purified PCR fragments and linearized pCB301 vector were mixed in proportion with 2×MultiF Seamless Assembly Mix (purchased from ABclonal, Wuhan, China), incubated at 50 °C for at least 30 min, and transformed into DH5α competent cells. Positive clones were screened by colony PCR using specific pCB301 vector detection primers (pCB301-det-F: CCTTCGCAAGACCCTTCCTCTA; pCB301-det-R: GACCGGCAACAGGATTCAATC) and sequenced commercially.

The pCB301-QMV plasmid was introduced into *Agrobacterium tumefaciens* strain GV3101 for agroinfiltration. The transformed agrobacteria were infiltrated into the abaxial surface of *Nicotiana benthamiana* seedling leaves and the cotyledon of mulberry leaves. For *N. benthamiana*, inoculated leaves were collected at 3 days post-infection (dpi), and systemic leaves (the second and third leaves at the top of inoculated leaves) were collected at 10 dpi. Cotyledon and true leaves were collected from mulberry seedlings at 10 and 20 dpi. The virus accumulation was detected by RT-PCR using QMV-RdRP primers (QMV-RdRP-F: CAAAGTTGAAAAGTACTCAGATGAAG; QMV-RdRP-R: TCCCTTGACCTTGATTTCGGAAC), and the reaction procedure was as follows: (1) 94 °C for 3 min; (2) 30 cycles, each consisting of 94 °C for 30 s, 55 °C for 30 s, and 72 °C for 1 min; and (3) 72 °C for 5 min. The PCR products were analyzed by electrophoresis on a 1.2% (*w/v*) agarose gel with nucleic acid dye (purchased from Takara, Beijing, China), and the visualization of DNA molecules was obtained using a BIO-RAD instrument (ChemiDoc XRS+, Bio-Rad, Hercules, CA, USA).

### 2.6. Electron Microscopy

The *N. benthamiana* and mulberry leaves were infected by QMV at 3 and 10 dpi. The infected leaf samples were cut into small pieces measuring 1 × 3 mm and fixed with 2.5% (*v/v*) glutaraldehyde and 2% (*v/v*) osmic acid, respectively. After each incubation for 2 h at room temperature, samples were washed three times with 0.1 M phosphate-buffered (PB) buffer.

After fixation, the plant samples were dehydrated in ethanol at 50%, 70%, 80%, 90%, and 95% (*v/v*) for 15 min. Then, samples were dehydrated with 100% (*v/v*) ethanol twice for 20 min, and with 100% (*v/v*) acetone all night.

The dehydrated samples were immersed in a mixture of spurr and acetone in the ratio of 1:1 (*v/v*), a mixture of spurr and acetone in the ratio of 3:1 (*v/v*) for 3 h, and in 100% spurr embedding agent all night. The sections to be cut were placed at the bottom of the tube and then polymerized at 70 °C for 24 h. Thin sections were subsequently cut and observed under a transmission electron microscope (8100, ZEISS, Shanghai, China) [28].

## 3. Results

### 3.1. Discovery of Viruses, Full-Length Genome Amplification, and Sequencing Results

**Figure 1:**
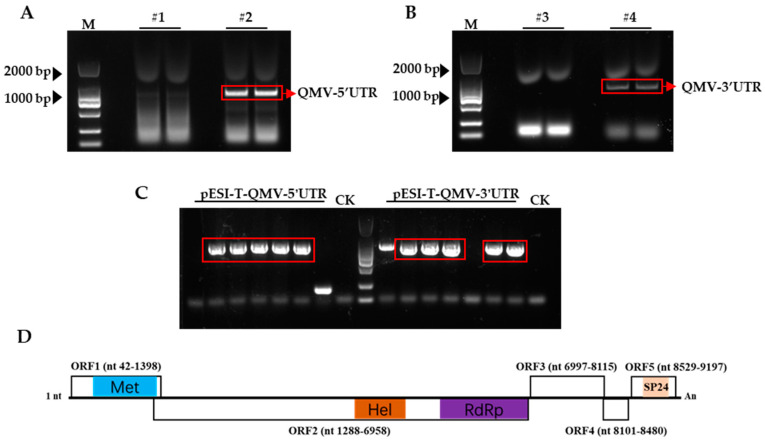
The result of RACE and the QMV gene structure. (**A**) The 5′-terminal PCR product of QMV. (**B**) The 3′-terminal PCR product of QMV. (**C**) The positive clone of pESI-T-QMV-5′UTR and pESI-T-QMV-3′UTR. (**D**) The putative open reading frames (ORFs, with boxes), conserved motifs, domains, and viral proteins (different colors). Viral methyltransferase (Met, pfam01660), viral helicase (Hel, pfam01443), RNA-dependent RNA polymerase_2 (RdRP, pfam00978), and SP24 (pfam16504). An: poly A tail.

We conducted a virome analysis by analyzing the transcriptome sequencing data from old mulberry leaves collected in the Kaiyuan Temple district of Quanzhou, Fujian Province (118°58′ N, 24°90′ E). Through this analysis, we discovered a new virus that infects mulberry trees, which we named Quanzhou mulberry virus (QMV), a potentially unclassified virus. To obtain the complete genome sequence of QMV, we utilized the RACE method. The 5′ and 3′-terminal PCR products were analyzed by electrophoresis on an agarose gel with nucleic acid dye. The visualization of DNA molecules is obtained using a BIO-RAD instrument (Figure 1A,B) and subsequently cloned into the pESI-T vector. We confirmed positive clones via colony PCR analysis (Figure 1C) and commercially sequenced them.

The QMV genome consists of 9256 nucleotides (nt) and includes five putative open reading frames (ORFs). ORF1, located at positions 3–1377 nt in the full-length genome, is predicted to encode a viral methyltransferase (Met, pfam01660, nt 375–1340, 322 aa) with a molecular weight of approximately 35.4 kDa. A blastx search of this sequence revealed the first three results to be 92% coverage and 29.93% sequence identity for Tohsystermes virus (QQM16332, unclassified Riboviria), 86% coverage and 31.99% sequence identity for Solenopsis invicata virus 17 (QRK69406, unclassified Iflaviridae), and 96% coverage and 29.31% sequence identity for Wuhan insect virus 8 (YP_009344994, unclassified Riboviria) (Table 1 and Table S1).

ORF2, located at position 1288–6993 nt in the full-length genome, is predicted to encode a viral RNA helicase (Hel, pfam01443, nt 4336–5106, 257 aa) and an RNA-dependent RNA polymerase 2 domain (RdRP_2, pfam00978, nt 5641–6939, 433 aa). The first three results from a blastx search were the following: 47% coverage and 31.31% sequence identity for Fasciogiga virus (DAZ87875, unclassified Riboviria), 46% coverage and 32.06% sequence identity for Beihai barnacle virus 2 (YP_009333216, unclassified Riboviria), and 48% coverage and 30.16% sequence identity for Cordoba virus (AQM55308, unclassified viruses) (Table 1 and Table S2).

The GenBank accession numbers used are as follows: Tohsystermes virus, QQM16332; Solenopsis invicta virus 17, QRK69406; Wuhan insect virus 8, YP_009344994; Fasciogiga virus, DAZ87875; Beihai barnacle virus 2, YP_009333216; and Cordoba virus, AQM55308. NS means no significant similarity sequences were found in the nr database.

ORF3 and ORF4 are located at positions 7015–8115 nt and 8113–8480 nt in the genome, respectively. The protein they encode is unknown. ORF5 is located at positions 8706–9104 nt in the genome and is predicted to encode a 133 aa SP24 protein (Figure 1). However, a Blastx search of the ORF3, ORF4, and ORF5 sequences did not yield any significant similarity with known proteins in the non-redundant (nr) database.

### 3.2. Phylogenetic Placement of QMV

**Figure 2:**
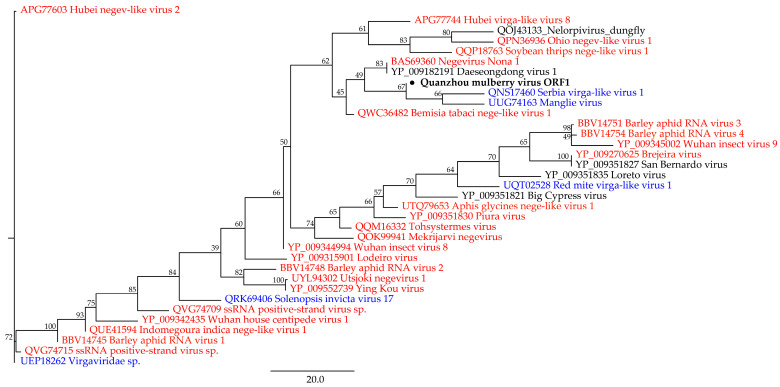
The phylogenetic tree displays Quanzhou mulberry virus ORF1, which has been marked and bolded. Members of unclassified Riboviria are represented in red, members of the kingdom Orthornavirae are represented in blue, and members of unclassified viruses are represented in black.

After conducting a blastx search on the full-length genomic sequence of QMV, it was found that 33.93% of the sequence showed similarity to unclassified viruses and 32.04% similarity to unclassified Riboviria. The first phylogenetic tree is based on the QMV ORF1 sequence and part of representative members, whose sequences were downloaded from the blastx search results. The phylogenetic analysis shows that the virus ORF1 falls in the clade with Daeseongdong virus 1 (YP_009182191, unclassified viruses) and Negevirus nona 1 (BAS69360, unclassified Riboviria, Figure 2).

**Figure 3:**
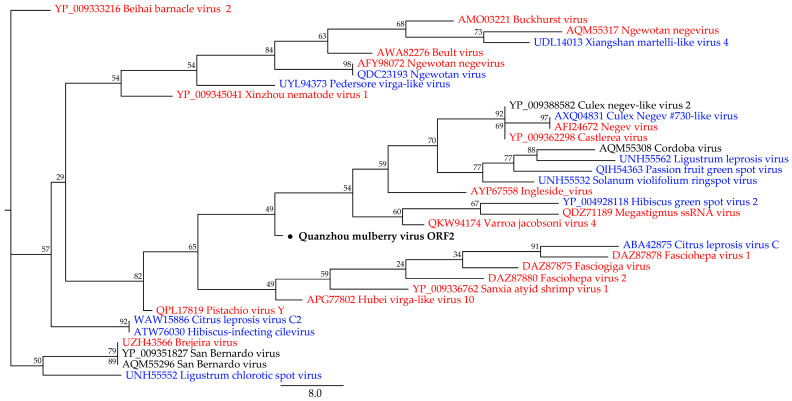
The phylogenetic tree displays Quanzhou mulberry virus ORF2, which has been marked and bolded. Members of unclassified Riboviria are represented in red, members of the kingdom Orthornavirae are represented in blue, and members of unclassified viruses are represented in black.

The second phylogenetic tree is based on the QMV ORF2 sequence and is partially representative of members downloaded from blastx search results containing RNA-dependent RNA polymerase. The phylogenetic analysis shows that the virus ORF2 is closely related to Varroa jacobsoni virus 4 (QKW94174, unclassified Riboviria), Megastigmus ssRNA virus (QDZ71189, unclassified Riboviria), and Hibiscus green spot virus 2 (YP_004928118, Higrevirus) (Figure 3).

Additionally, when performing a blastx search on the ORF3, ORF4, and ORF5 sequences, no significant similarity with known proteins in the nr databases was found. Based on these findings, it can be concluded that QMV should be classified as a member of the unclassified Riboviria.

### 3.3. QMV Infectious Clone Construction and Infectivity in Nicotiana benthamiana

**Figure 4:**
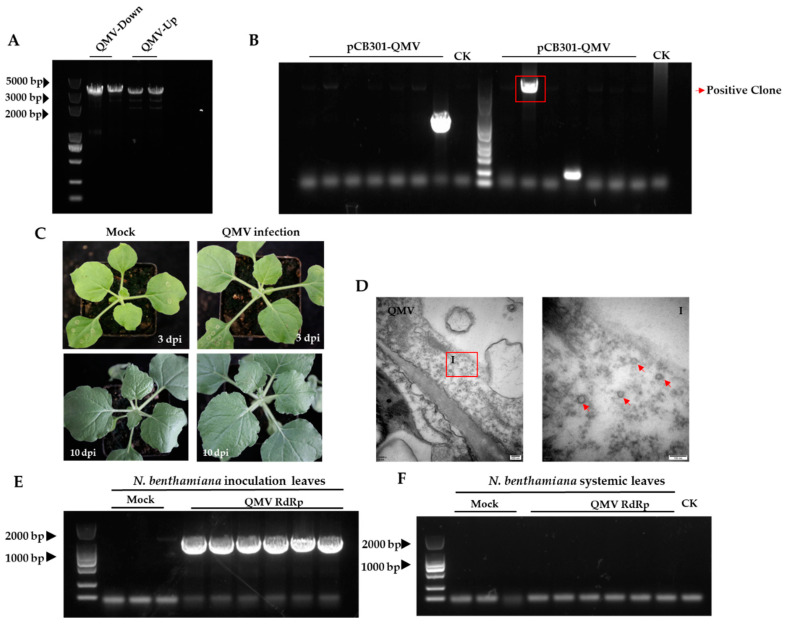
QMV infectious clone construction and typical signs of Quanzhou mulberry virus (QMV) infected *N. benthamiana*, virion observation, and virus detection. (**A**) Two fragments PCR products from QMV full-length genomic RNA. (**B**) The positive clone for pCB301-QMV. The red box is the destination strip. (**C**) Symptoms of QMV infection in *N. benthamiana*. (**D**) Electron micrographs of virions in QMV-infected *N. benthamiana* leaves. I panels are the enlarged images of the red boxed areas I in the above panels. Red arrows indicate a QMV virion in the cytoplasm of *N. banthamiana*. (**E**) QMV RdRP detection for inoculated leaves from *N. benthamiana*. (**F**) QMV RdRP detection by RT-PCR in the apical leaves of *N. benthamiana*.

To evaluate the infectivity of QMV, two fragments covering the QMV sequence were amplified and cloned into the pCB301 vector. The resulting PCR products were analyzed by electrophoresis on a 1.5% (*w/v*) agarose gel with nucleic acid dye (Figure 4A), and the purified fragments were seamlessly cloned into the linearized pCB301 vector. Positive clones were screened by colony PCR (Figure 4B) and subsequently sequenced to confirm their identity.

The recombinant agrobacteria were then infiltrated into the abaxial surface of *N. benthamiana*. As a result, asymptomatic infections were observed on both inoculated leaves and systematic leaves (Figure 4C). Electron micrographs revealed the presence of regular icosahedral QMV virions distributed in the cytoplasm of the inoculated leaves (Figure 4D). RT-PCRs were performed using QMV RdRP-specific primers for the inoculated leaves at three days and systematic leaves at ten days. The virus was detected in the inoculated leaves but not in the systemic leaves (Figure 4E,F).

### 3.4. QMV Infectivity in Mulberry

**Figure 5:**
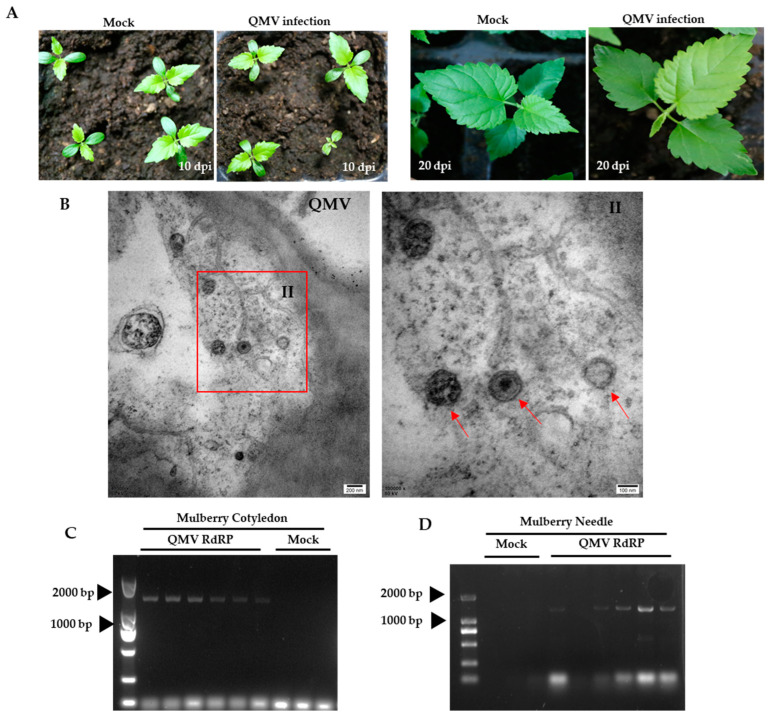
Typical signs of Quanzhou mulberry virus (QMV)-infected mulberry, and virus detection. (**A**) Symptoms of virus infection at different times for mulberry. (**B**) Electron micrographs of virions in QMV-infected cotyledons of mulberry. Panels II are the enlarged images of the red boxed areas II in the above panels. Red arrows indicate a QMV virion. (**C**) QMV RdRP detection for cotyledon from mulberry. (**D**) QMV RdRP detection for true leaves from mulberry.

The inability of QMV motion from infected leaves to systemic leaves in *N. benthamiana* is probably because it was not its natural host. Therefore, agrobacteria carrying the QMV infectious clone were infiltrated into the abaxial surface of mulberry cotyledons. No symptoms were observed on either the cotyledons or true leaves of infected mulberry seedlings (Figure 5A). Electron micrographs revealed the presence of regular icosahedral virions in the leaves of mulberry seedlings (Figure 5B). To confirm the presence of the virus, RT-PCR was performed using QMV RdRP-specific primers on both cotyledons at ten days and true leaves at twenty days after infiltration. The results show that the virus was detected in both the cotyledons and true leaves (Figure 5C,D), suggesting that QMV can achieve systemic infection in mulberry. This finding indicates that QMV may have a species-specific host range.

## 4. Discussion

This study presents the identification and characterization of a new virus, Quanzhou mulberry virus (QMV), using transcriptome sequencing of mulberry leaves collected in Kaiyuan Temple, Quanzhou, Fujian Province, China. Phylogenetic analysis revealed QMV as an unclassified Riboviria. The complete QMV genome was successfully amplified, and an infectious clone was generated. Agroinoculation assays were used to investigate QMV infectivity in *N. benthamiana* and mulberry seedlings, with asymptomatic infections in both plants. The results of RT-PCR detection found that QMV virus was accumulated in the inoculated leaves of both *N. benthamiana* and mulberry, but only in the apical leaves of mulberry. This suggests that QMV may have a species-specific host range. Our findings provide valuable insights into the pathogenesis and host tropism of QMV.

Based on QMV biological properties, developing a QMV vector for expressing foreign genes in mulberry appears feasible. In recent years, several virus vectors have demonstrated successful use in expressing foreign genes for controlling and researching gene function in fruit trees. Previous studies on virus vectors have shown their ability to effectively express foreign proteins. For instance, since citrus leaf blotch virus (CLBV) infections are symptomless in most citrus species, CLBV-based vectors were successfully used to analyze citrus gene function by silencing or overexpressing these genes in citrus trees [18,29,30]. CLBV-based vectors were also used to express the Arabidopsis thaliana or citrus Flowering Locus T (FT) genes in citrus, and as a result, the juvenile period of citrus trees has been reduced from 6 years to 4–6 months. More importantly, flowering was observed for a minimum of 5 years, indicating that these vectors are highly stable [18,29,30]. Similarly, the apple latent spherical virus (ALSV), a common virus infecting apple, pear, and cherry trees without showing obvious symptoms, has emerged as a potential vector for expressing or silencing foreign sequences in apple and pear trees, and the phenotypes could continue for at least several months [31,32]. The potential of QMV as a vector for expressing foreign genes in mulberry warrants further investigation, as it could have important implications for mulberry research and production.

Previous studies have proposed that weakened strains of plant viruses can be utilized in viral biocontrol techniques to protect plants from more severe strains of the same species [33]. The application of Oryctes virus into areas experiencing an outbreak of rhinoceros beetle (*Oryctes rhinoceros*, Coleoptera: Scarabaeidae) has been a successful example of classical biocontrol with a virus, resulting in a significant reduction in palm damage in numerous regions throughout the Asia/Pacific area [34]. Similarly, the mild mutant of type P Hawaii severe strain (PRSV P-HA), Papaya ringspot virus (PRSV) HA5-1, has been extensively used to control PRSV type P strains in papaya [35]. Given the infectivity and pathogenicity of QMV, it is possible that this virus could be utilized as a biological control agent. However, further research is required to determine whether QMV could be employed as a biocontrol agent for mulberry or other plant species.

Transcriptome sequencing and infectious clone technology have been valuable tools for discovering and characterizing novel plant viruses. Further research into the ecology, evolution, and molecular biology of plant viruses will be essential for understanding their impact on plant health and global food security. The differing abilities of QMV to move within mulberry and *N. benthamiana* plants suggest specific interactions between the virus and its plant hosts, which could be further investigated for important insights into the interactions between QMV and its plant hosts. Overall, this study expands our knowledge of the diversity and evolution of plant viruses and provides valuable tools and insights for future investigations into QMV and related viruses.

## Figures and Tables

**Table 1:** The summarized BLAST results of QMV ORF.

	Blastx Results	Coverage	Identity	Taxonomy
QMV ORF1	Tohsystermes virus	92%	29.93%	unclassified Riboviria
	Solenopsis invicta virus 17	86%	31.99%	unclassified Iflaviridae
	Wuhan insect virus 8	96%	29.31%	unclassified Riboviria
QMV ORF2	Fasciogiga virus	47%	31.31%	unclassified Riboviria
	Beihai barnacle virus 2	46%	32.06%	unclassified Riboviria
	Cordoba virus	48%	30.16%	unclassified viruses
QMV ORF3	NS			
QMV ORF4	NS			
QMV ORF5	NS			

## Data Availability

The supplementary data related to this paper are included. Raw data can be obtained by request. The complete QMV sequence and raw sequence data presented in this paper have been deposited at the National Center for Biotechnology Information (NCBI, https://www.ncbi.nlm.nih.gov/ (accessed on 6 April 2023)). The sequence of QMV has been assigned the GenBank accession number OQ829350, while the SRA accession numbers are PRJNA952904.

## Supplementary Materials

The following supporting information can be downloaded at: https://www.mdpi.com/article/10.3390/v15051131/s1. There are three supplementary tables. Table S1: The blastx result of the QMV ORF1 sequence; Table S2: The blastx result of the QMV ORF2 sequence; File S1: The whole sequence of QMV.
